# Churchill’s Midwifery

**Published:** 1848-05

**Authors:** 


### CHURCHILL'S MIDWIFERY.

A new edition of this work has just been issued by Messrs. Lea & Blanchard, which contains the additions rendered necessary by the progress of obstetrical science. It is decidedly a favorite with the American profession, and the present edition, no doubt, will meet with as much approbation as the two which have been exhausted.

